# The Oldest Art

**DOI:** 10.3201/eid3105.AC3105

**Published:** 2025-05

**Authors:** Byron Breedlove

**Affiliations:** Centers for Disease Control and Prevention, Atlanta, Georgia, USA

**Keywords:** fungi, fungal infections, art-science connection, Adolphe Philippe Millot, *Champignons-couleurs 2*

**Figure:**
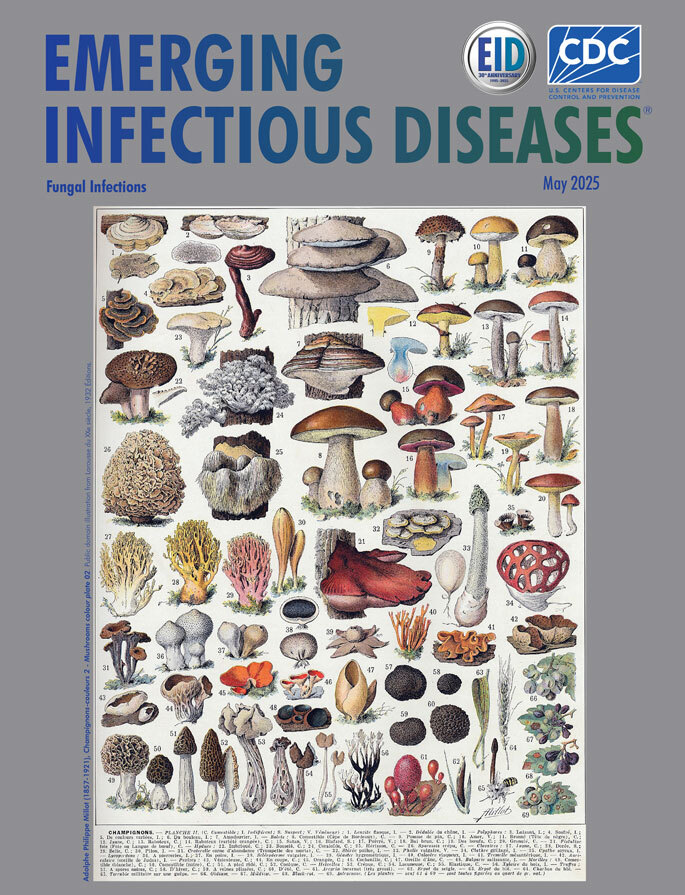
**Adolphe Philippe Millot (1857–1921). *Champignons-couleurs 2* (*Mushrooms color plate 02*)*.*** Public domain illustration from Larousse du XX^e^ siècle, 1932 Éditions.

“Nature alone is antique, and the oldest art a mushroom” is an enduring quote from Scottish historian and writer Thomas Carlyle. This month’s cover image, a color plate titled *Champignons—*the French word for mushrooms—from the 1932 edition *Larousse du XX^e^ siècle* (*Larousse of the 20th century*), celebrates examples of “the oldest art.” Developed under the direction of editor and lexicographer Paul Augé, this six-volume *Larousse* comprised an estimated 235,000 articles, spanned 6,523 pages, and served as an encyclopedia and dictionary of the French language.

This natural history plate is the handiwork of Adolphe Philippe Millot, a painter, lithographer, and entomologist. Details about Millot’s life are scant. Historian and curator William B. Ashworth notes that Millot “was the senior illustrator at the Museum of Natural History in Paris, at the end of the 19th century and the beginning of the 20th. But that’s all we know. He presumably had a family, knew many French naturalists who *are* well known, and perhaps even had a certain social standing.” Ashworth adds, “Millot went on to do thousands of illustrations for the *Larousse Encyclopedias* that appeared in various forms in the early 20th century.”

Contemporary viewers will quickly recognize—before consulting the minuscule type in the figure’s legend—that this colorful compendium includes fungi other than mushrooms. During Millot’s lifetime (and for nearly another half century), fungi were thought to be plants, and classifying fungal species was a nascent endeavor. The spell of Swedish botanist and taxonomist Carl Linnaeus, who had concluded in the mid-1700s that living organisms were either plants or animals, still lingered. During the 1960s, American plant ecologist Robert Whittaker determined that fungi are not plants and devised a taxonomic classification of the world’s biota into five kingdoms: Animalia, Plantae, Fungi, Protista, and Monera.

Fungi are strong contenders for being the earth’s least understood organism. Recent molecular studies demonstrate that fungi are more closely related to animals than to plants and fundamentally differ from plants at the cellular level. Cell walls of fungi are composed of chitin, also found in the exoskeletons of insects and crustaceans and in squid beaks, whereas cell walls of plants are made from cellulose. Fungi absorb nutrients from soil or other organic sources, such as rotting trees, whereas plants make their own food through photosynthesis. There is no decisive estimate on the number of fungi in existence. For instance, the authors of a 2024 article in the journal *IMA Fungus* estimated the number of fungal species to be between 1.5 and 10 million.

Science writer Cody Cottier offers this perspective: “Point to a patch of dirt, a body of water, even the air you’re breathing, and odds are that it is teeming with mushrooms, molds and yeasts (or their spores) that no one has ever seen. In ocean trenches, Tibetan glaciers and all habitats between, researchers are routinely detecting DNA from obscure fungi. By sequencing the snippets, they can tell they’re dealing with new species, thousands of them, that are genetically distinct from any known to science. They just can’t match that DNA to tangible organisms growing out in the world.”

Humans have found myriad uses for fungi, including as food; nearly 2,200 species of mushrooms can be safely consumed, and an estimated 100 species of known mushrooms can poison people. Fungi function as a leavening agent for breads; enable fermentation of cheeses and olives; and convert sugars from grains and fruits into beer, cider, and wine. At least 300 species of mushrooms contain psychoactive components—something indigenous cultures across the world have known for centuries—and contemporary clinicians and therapists are using such compounds for treating depression, posttraumatic stress disorder, substance abuse, and chronic pain. Antibiotics developed from fungi, such as penicillin and cyclosporine, have saved untold lives.

On the other hand, fungi also sicken and kill people. Hundreds of species of fungi, enough to fill perhaps 10 of Millot’s color plates, are known to cause infections in humans. The Centers for Disease Control and Prevention notes that more than 1 billion people around the world experience a fungal infection each year. Aspergillosis, blastomycosis, candidiasis, coccidioidomycosis, cryptococcosis, histoplasmosis, and mucormycosis are fungal diseases that pose serious health concerns and are potentially deadly.

Microbiologist Arturo Casadevall explains, “Fungal diseases are difficult to manage because they tend to be chronic, hard to diagnose, and difficult to eradicate with antifungal drugs.” Climate change, antimicrobial resistance, and various other factors are likely to lead to the emergence of fungal infections.
